# 

**Published:** 1822-03-01

**Authors:** 


### BIBLIOGRAPHICAL RECORD;

OR

*Works received for Review, within the Quarter.*

1. Thoughts and Suggestions relative to the Cause, Nature, and Treatment of Intestinal Inflammation, including an Affection to which Lying-in-women are subject. By a Philanthropist. Dublin, 1820. 8vo. pp. 42

☞ *We are much obliged by the unknown Author's present of the above, and also the polite note which accompanied it.*

2. A Medical Guide to the Cheltenham Waters, containing Observations on their Nature and Properties; the Diseases in which they are beneficial or hurtful; with the Rules to be observed during their Use. By William Gibney, M.D. one of the Physicians to the Cheltenham Dispensary, &c. Small 8vo. pp. 162. Cheltenham. 1821.

---

3. On the Re-establishment of a Canal in the Place of a Portion of the Urethra which had been destroyed. By Henry Earle, Esq. Surgeon to the Foundling, and Assistant Surgeon to St. Bartholomew's Hospital. Quarto, pp. 14. From the Philosophical Transactions. London, 1821.

---

4. On the Nerves; giving an Account of some Experiments on their Structure and Functions, which lead to a new Arrangement of the System. By Charles Bell, Esq. Quarto, pp. 29. From the Philosophical Transactions, 1821.

---

5. Notes on the Medical Topography of the Interior of Ceylon; and on the Health of the Troops employed in the Kandyan Provinces, during the Years 1815-16-17-18-19, and 1820: with brief Remarks on the prevailing Diseases. By Henry Marshall, Surgeon to the Forces. Octavo, pp. 228. London, 1821.

---

6. A Treatise on Diseases of the Nervous System. *Part the First.* Comprising Convulsive and Maniacal Affections. By J. C. Prichard, M. D. Late of Trinity College, Oxford; Fellow of the Linnæan and Wernerian Societies, &c. Physician to St. Peter's Hospital and the Bristol Infirmary. One volume, 8vo, pp 425. London, 1822.

---

7. The Introductory Lecture of a Course upon *State Medicine*, delivered in Mr. Grainger's Theatre, Southwark, on Thursday, November 1st, 1821. By John Elliotson, M. D. &c. &c. Octavo, pp. 35. Longman and Co. London, 1821.

☞ *This introductory Lecture appears to be well calculated to impress on the young mind the necessity of studying forensic medicine as an essential part of a regular medical education. We wish Dr. Elliotson every success in his laudable endeavours to disseminate medico-legal knowledge throughout the rising generation.*

---

8. An Essay on the Effects of the Fucus Helminthocorton upon Cancer, more especially in the stage denominated Occult; with a concise Enquiry into the Origin and Nature of the Disease, and the probable way in which the Dispersion of the Tumour is effected, &c. &c. By William Farr, Member of the Royal College of Surgeons, London; and late Surgeon to the Hospital on the Island of Anholt. One vol. 8vo, pp. 112, with a Plate of the Fucus. London, 1822.

---

9. Considerations sur une Alteration Organique appelé Digenerescence Noire, Melanose, Cancer Mélané, &c. Par G. Breschet, Chef des travaux Anatomiques à la Faculté de Medecine de Paris, &c. &c. Octavo, pp. 24, with one coloured Plate representing the Melanose. Paris, 1821.

10. The Philadelphia Journal of the Medical and Physical Sciences. Edited by Dr. N. Chapman. No. 5, November, 1821.

---

11. The American Medical Recorder, of original Papers and Intelligence in Medical Science. No. XV. for July, and No. XVI. for October, 1821.

---

12. A final Reply to the numerous Slanders circulated by N. Chapman, M. D. By Granville Sharpe Pattison. Octavo, Second Edition. Baltimore, 1821.

☞ *We hope this will prove a finale to those acrid controversies and personal contentions which we have been sorry to observe among our American brethren Let a stranger, but a well-wisher, in the "old world" recommend a cessation of these hostilities in the land of liberty and independence. They occasion, at the best, but a momentary and painful gratification, even in the breast of the victor.—They invariably bring after them contrition and remorse in the minds of both parties.*

---

13. Reflections sur les Fievres, Par J. B. G. Barbier, Professeur à l'Ecolè Secondaire de Medecine d'Amiens, &c. Octavo, Paris, 1821.

---

14. Revue Medicale Historique et Philosophique, Nos. for September and October, 1821.

---

15. Della Maniera più atta a Curare Radicalemente le varici ad Impiagamenti varicosi dell'Estremità inferiori. Memoria di Ranieri Cartoni Medico-Chirurgo. Pisa, 1821.

☞ *We are much obliged to our esteemed friend, Dr. Clark, of Rome, for this publication.*

---

16. Cases illustrative of the Treatment of Diseases of the Ear, both local and constitutional, with Practical Remarks relative to the Deaf and Dumb. By John Harrison Curtis, Esq. Aurist to His Majesty, &c. &c. &c. Octavo, pp. 93. London, 1822.

☞ *These cases are fifty-eight in number, detailed with more or less minuteness, and designed to illustrate the principles laid down in Mr. Curtis's Treatise on the Ear. They are therefore totally incapable of analysis. The treatment, in general, appears to be judicious, simple, and successful. The principal items in the methodus medendi are, syringing the ear frequently—the application of detergent or gently stimulating ointments—blisters—leeches—lunar caustic—unguentum hydrargyri nitratum, &c. To these local means Mr Curtis frequently adds alterative doses of calomel or blue pill, with a cathartic twice a week. We have no doubt that, if the above means are judiciously adapted to the existing indications in each case, they will insure as much success as can reasonably be expected in the present state of our knowledge.*

---

17. Memoire sur le Vomissement considéré dans l'Etat Sain et dans les Maladies cancereuses de l'Estomac. Par M. Piedagnel, Interne de Premiere Classe des Hopitaux et Hospice Civile de Paris, Prosecteur à l'Athenée Royale. Paris, 1821. Octavo, sewed, pp. 29. *From the Author through Mr. Carpue.*

☞ *A very ingenious little essay confirming the new doctrine of M. Magendie respecting the action of vomiting. We shall make some extracts from it in our next number.*

---

18. Medicamina Officinalia, seu Pharmacopœia Londinensis, Index Methodicus. Cura F. A. Macann, M. D. Londini, 1822, pp. 92. Duodecimo.

☞ *The plan of this little index is, to exhibit all the officinal simples in one continued alphabetical series, and to enumerate under each simple, all those articles which are, in any manner, derived or formed from it. It thus forms a kind of analytical view of the Pharmacopœia, which may be usefully appended to that work. Dr. Macann has also added notes, critical and explanatory, which may be perused with advantage.*

---

19. An Epitome of Pharmaceutical Chemistry, whereby the Art of prescribing scientifically may be facilitated, and those decompositions avoided, which, resulting from combinations of incompatible substances; often frustrate the views of the practitioner in their medical effects; arranged according to the London Pharmacopœia. By Rees Price, M. D. Member of the Royal College of Surgeons in London; Honorary Member of the Medical and Physical Society of Guy's Hospital; Member of the University of Heidelberg, &c. Duodecimo, pp. 59, 1822.

---

20. A Chart of Pharmaceutical Chemistry; exhibiting the Names of the various Articles of the London Pharmacopœia, in contrast with those with which they are incompatible; whereby the art of prescribing scientifically may be facilitated, and those decompositions avoided, which often frustrate the views of the Practitioner in their medical effects. By Rees Price, M. D. &c. &c. &c. Price 2s. London, 1822.

☞ *This Epitome and Chart, especially the latter, will be found useful for reference in extemporaneous prescription, and ought to be in the surgery or the dispensary of the medical practitioner. The author appears indebted for almost the whole of his compilation to the works of Dr. Paris, and Mr. A. T. Thompson; still the decompositions being brought into a single point of view, are more easily referred to than in the sources above-mentioned.*

---

21. A Treatise on Indigestion and its Consequences, &c. &c. By A. P. W. Philip, M. D. *Second Edition, with some additional Observations.* London, 1822.

---

22. Essays on Surgery and Midwifery; with Practical Observations and select Cases. By James Barlow, Surgeon. With Plates. One vol. 8vo, pp. 417. London, 1822.

☞ *This appears to be a valuable work, of which we shall give an account in our next number.*

---

23. The Principles of Medicine, on the Plan of the Baconian Philosophy. Vol. I. on Febrile and Inflammatory Diseases. By R. D. Hamilton. Octavo, pp. 295. London, 1822.

24. An Essay on the Symptoms and History of Diseases; considered chiefly in their Relation to Diagnosis. By MARSHALL HALL, M. D. F. R. S. E. and formerly Senior President of the Royal Medical Society of Edinburgh. Octavo, pp. 130, very small type. London, 1822.

*This is a re-publication, considerably improved, of the* FIRST *part of the author's work on Diagnosis. As the work has never been analyzed in the quarterly form of our Journal, we hope to be able to give a particular account of this second edition in our next number.*

---

25. A Manual of the Climate and Diseases of Tropical Countries; in which a Practical View of the Statistical Pathology and of the History and Treatment of the Diseases of those Countries is attempted to be given: calculated chiefly as a Guide to the young Medical Practitioner on his first resorting to those Countries. By COLIN CHISHOLM, M. D. F. R. S. &c. &c. &c. One volume, 8vo, closely printed, pp. 336, with a Plate. London, 1822.

☞ *This most excellent Manual we conscientiously recommend to every one destined for the Torrid Zone. In our next we will give a detailed account of the work.*

---

26 A Letter to Charles Henry Parry, M. D. F. R. S &c. &c. on the Influence of Artificial Eruptions in certain Diseases incidental to the Human Body; with an Inquiry respecting the probable Advantages to be derived from further Experiments. By EDWARD JENNER, Esq. M. D. LL. D. F. R. S. &c. Physician Extraordinary to the King. Quarto, pp. 67. Baldwin, London, 1822.

☞ *A full account of this Letter in our next.*

---

27. The New England Journal of Medicine and Surgery, &c. Nos. III. and IV. New Series, July and October, 1821.

---

N. B. Authors and Publishers will readily perceive the great importance of having their works regularly registered, with full titles, in this department, which stands with the Journal itself, and forms a perpetual advertisement. They will therefore be careful to have copies sent as soon as possible after publication.

### APPENDIX, No. 1,

TO THE

### GENERAL LIST OF SUBSCRIBERS IN VOL. I.

BEING

### *THE ADDITIONAL SUBSCRIBERS*

DURING THE LAST TWELVE MONTHS.*

A.

Acton, Mr. Surgeon, Ludlow
Allen, Oswald, Esq. York
Anderson, Mr. Surgeon, R. N.
Andrew, Mr. Surgeon, Fetter Lane.
Asled, Mr. Surgeon, Halifax
Attree, William, Esq. Surgeon, Brighton

B.

Babington, Dr. Aldermanbury
Bailey, Mr. Charles, Kirkleatham, Yorkshire
Bailey, Mr. H. W. Thetford
Baker, William, Esq. Charlotte-street, Bedford-square
Baltimore Medical Society, United States
Barbadoes Military Medical Society
Bartlett, Mr. Surgeon, Great Bedwin
Bateman, Geo. Esq. Surgeon, Yarmouth
Baxton, Mr. Surgeon, Brighton
Beck, Mr. Edward, Jesus College, Cambridge
Berth, Mr. James, Surgeon, Halifax, Yorkshire
Billet, Thomas, Esq.
Birdsall, William, Esq. Surgeon, Syston, Leicestershire
Black, Dr. J. Bolton, Lancashire
Black, Mr. Surgeon, Back-road Islington
Bloomfield, Dr. Woodbridge
Bonsfield, Dr. Spilsby
Bowden, Stephen, Esq. Surgeon, R.N. Plymouth
Boyle, Mr. James, late Surgeon H. M. S. Minden
Bradley, Mr. Joseph, Mytholm Royd, near Halifax, Yorkshire
Brewster, James, Esq. Tolling-hunt, Darcy
Bridget, Sam. M. D. Honorable Company's Service
Brown, Mr. Surgeon, Brighton
Buckley, Mr. Surgeon, Thursk
Bull, Cristopher, M. D. Cork
Burder, Dr. King's-road, Bedford Row
Burke, Dr. Pembroke

C.

Caldwell, Charles, M. D. Dean of Transylvania University, Lexington, Kentucky
Campbell, Dr. Dougall, Surgeon, Royal Artillery, Member of the Royal Physical Society of Edinburgh, at present resident Physician (by permission of the King of France) at Boulogne, Sur Mer

* A few names accidentally omitted in the last *general* list having come too late in the last quarter of the year, are here inserted. A general list of the *whole* subscribers will be printed at the end of every third or fourth volume.

Cample, John, M. D. Surgeon, Ashton-under-line
Carrich, Mr. Surg. Kennington
Carruthers, Mr. Surgeon, Romford
Chester Medical Society
Christie, Robert, Esq. Hon. East India Company's Service
Christie, Mr. Spencer-street, Northampton-square
Clarke, Mr. Surgeon, Lincoln's-Inn-fields
Coates, Mr. Surgeon, Haydon Bridge, Hexham
Cochran, Dr. Madras Establishment
Coley, Dr. R. W. Resident Physician, Cheltenham
Cooke, W. Esq. Dover
Cooper, Mr. Charles, Surgeon, Upper Tooting
Cooper, Dr. Dorchester
Collins, John, Esq. Surgeon
Corfu Garrison Medical Library
Cork Institution
Coyne, Doctor, Sligo, Ireland
Crisp, J. B. Esq. Surgeon, Brixworth, Northamptonshire
Crosley, Mr. Surgeon, (place not stated)
Cross, Mr. John, Surg. Norwich

D.

Darling, Dr. Brunswick-square
Davids, Mr. Surg. West Cowes, Isle of Wight
Davies, Mr. T. L. O.
Davies, Dr. W. A. Tenby
Davis, Dr. St. Kitts
Davis, Mr. Surgeon, Andover, Hants
Daubeny, C. (M. D.) Oxford
Dawson, Messrs. Surg. Wakefield, Yorkshire
Deal Medical Society
Dempster, Mr. John, Assistant Surgeon, Cape of Good Hope
Dennet, John Thomas, L. S. A. Surgeon, Bognor
De Sanctis, Dr. London
Deverill, G. S. Esq. at Dr. Ballingall's Howe-street, Edinb.
Diac, Mr. James, M. Leek
Dickinson, Mr. Josiah, Croxton, near Chorley, Lancashire
Dickenson, Mr. Wigmore-street
Dickson, Mr. Surgeon, H. M. S. Carnation, West Indies
Dill, John, Esq. Surgeon, East India Company's ship Atlas
Dill, Dr. Marquis, Newton Limavady, Ireland
Dillon, Charles Esq.
Dingle, Mr. Bookseller, Bury, six copies
Doak, Michael, Esq. Surg. R.N.
Douglas, John, M. D. New South Wales
Drake, Dr. Hadleigh, Suffolk
Duke, Dr. Valentine, late Surgeon to His Majesty's Naval Hospital, Cape of Good Hope
Dumfries Medical Society
Duncan, Mr. Surgeon, Wrotham

E.

Eachus, Mr. George, Surgeon, Saffron Walden, Essex
Editors of Edinburgh Medical and Surgical Journal, in exchange for Med. Chir. Rev.
Edwards, Henry, Esq. Bartholomew's Hospital
Erskine, William, Esq. Bombay Establishment
Evans, Mr. Surgeon, Belper, Derbyshire

F.

Faculty of Physicians and Surgeons, Glasgow
Faulkner, Sir Arthur Brooke, M. D. Cheltenham
Ferguson, Hugh, Esq. Surgeon, R. N.
Fergusson, Mr. R. N.
Field, Henry, M. D. Black-rock near Dublin
Filkin, Dr. Thomas
Fitzgerald, Mr. Percy-street
Fouler, Mr. Edward

Flemming, John, Esq. M. P. Gloucester-place
Foulkes, Ed. Esq. Surg. Ruyton, (Eleven Towns) Salop
Fraser, Dr. Doughty-street
Freeman, Mr. Surg. Bracknell, Berks
Freeman, Joseph, Esq. Spring Gardens
Freeman, R. Esq. Saxmundham
Freers, W. F. Esq. Surgeon, Stourbridge
Furber, G. Esq. Memb. Royal Coll. Surg. Malpas, Cheshire

G.

Gannon, Mr. John, Assist. Surg. H. M. S. Medina
Gediké, Dr. C. E. Berlin
George, Henry, Esq. Surgeon, Kensington
Gilbert, Mr. J. B. Surg. Chapel Row, Worthing
Gibney, Dr. Wm. Cheltenham
Gibson, Mr. Erasmus
Godfrey, Mr. Surg. Chenlbury, Oxfordshire
Godwin, Mr.
Goldie, Dr. George, York
Golding, Benj. Esq. Surgeon, St. Martin's Lane
Goodwin, Mr. Surgeon, Hon. E. I. Company's Service
Grady, Mr. C. (place not stated)
Graham, Mr. Surgeon, Broad-street, Golden-square
Granger, B. Esq. Surgeon, Burton-on-Trent
Griffiths, Dr. Royal Navy
Griffith, Dr. Philadelphia
Grigg, Mr. Surgeon, R. N.
Gwyn, R. H. Esq. Surgeon, Broseley, Salop

H.

Hammond, Mr. C. C. Surgeon, Ipswich
Hardy, Dr. C. H. Fellow of the Royal College of Physicians, Bath
Harris, Mr. Surgeon, Mark-lane
Harsant, Mr. C. Wrickham Munket
Hart, William, Esq. Surgeon, Dorking, Surrey
Herberski, Profes. Vincent, University of Wilna, Lithuania
Hey, Mr. Surgeon, Kenninghall
Hill, Mr. Ninian, Greenock
Houghton, Mr. Conduit-street
Howel, Mr. J. Surg. Wandsworth
Huggins, Mr. Surgeon, Derby
Hulke, William, Esq. Deal
Hunter, Dr. Adam, Leeds
Hutchisson, Dr. Guernsey

I. J.

Illingworth, Mr. Surgeon, Bradford, Yorkshire
Ireland, Mr. Surgeon, Hart-street, Bloomsbury
Irvine, Mr. S. Surgeon, H. M. S Sophy, to East Indies
Ivory, Mr. Surg. Nassau-street
Jackson, Mr. (place not stated)
Jackson, Robert, M. D. Stockton on Tees
Jackson, Mr. T. D.—To East Indies
James, Dr. D. Bourdeaux
Jefferson, Mr. T. R. Surg. Middlesex-place, New-road
Jice, Mr. Surgeon, Ware
Johnson, Mr. at London Hospital
Johnstone, J. C. Esq. Member of the Royal College of Surgeons of London, His Majesty's Ship Requin, Grenada, West Indies
Jones, Mr. Henry, Surgeon, Welsh-Pool

K.

Keddel, Mr. Surgeon, Deptford
Keegan, Dr. Halifax, Nova Scotia
Kenny, Mr. (Student)
Keys, Thomas Esq. Assistant-Surgeon, Madras
Kinderwood, Mr. Surgeon-Accoucheur to the Manchester Lying-in-Hospital

King, R. C. Esq. Saxmundham
Knight, Mr. Paulslade, Governor of Lancaster County Asylum

L.

Laird, Dr. Physician to Guy's Hospital, Bloomsbury-square
Langstaff, Mr. W. H. Surgeon, Stockton
Lara, Benj. M. D. of the Royal College of Physicians, Ed. Resident Physician, Portsea, Hants
Leeds Medical Society
Lexington Library, Kentucky, America
Lloyd, W. M. D. Chatham
London, J. Esq. Surgeon of His Majesty's Ship Doris
Loxley, G. Esq. Member of the Royal College of Surgeons, Rotherane
Lucas, Dr. Brecknock
Lynn, G. D. Esq. Woodbridge

M.

Mac Cabe, Dr. London
M'Carthy, Dr. A. Skibbereen, Cork
M'Corkell, Isaac, Esq. Surgeon, Jamaica
M'Crea, Dr. James, Royal Navy
Macdowal, Mr. Surgeon, Bengal
Mackintosh, Dr. R.D. (late Physician to the Essex and Colchester Hosp.) Queen Square, Bloomsbury
M'Lean, Mr. Joseph, Surgeon of His Majesty's Ship Medina, Halifax
Maclean, Allan, M. B. one of the Physicians to the Essex and Colchester Hospital
Maclean, Sir L. Physician, Sudbury, Suffolk
M'Manus, Mr. R. Paris
Macmichael, Dr. Fellow of the Royal College of Physicians, Albany, Piccadilly
M'Millan, Dr. Deputy Inspector Army Hospitals
Magendie, Dr. Paris
Major, T. Esq. Surgeon, Hungerford, Berks, Member of the Royal College of Surgeons, and Society of Apothecaries, and late Surgeon in His Majesty's Navy
Manley, Dr. James R. New York, (in exchange)
Mann, George, Esq. Surg. Cork
Mansel, Thomas, Esq. Surgeon, Pembroke
Martin, Dr. Peter, Dunning, Perthshire
Manigy, Daniel Pallot, Esq. Guernsey
Maxton, Mr. James, Surg. Crief
Medical Library, General Infirmary, Northampton
Miller, Dr. E. Londonderry
Mitchell, Mr. Thomas, Member of Royal College of Surgeons, and Licentiate of the Apoth. Company, Whitehaven, Cumberland
Morris, Mr. Thomas, Surgeon, Ashton-under-line
Morton, Mr. Chas. Londonderry
Murray, Mr. Surgeon, Frome

N.

Nash, Mr. James, Surgeon, Hartleberry, Worcestershire
Newman, Mr. Surgeon, Mere, Wilts
Noble, Mr. Martin Shelston
Norman, Charles James, Esq.—To West Indies
Northen, Dr. Newcastle-under-line

O.

O'Donnel, Dr. 4th Dragoons, Bombay
Osborne, Alick, Esq. H. M. S. Leven—To West Africa
Owen, W. Esq. Surg. Glandrilay, near Llanidloes, Wales

P.

Padmore, Mr. Charles Henry, Surgeon, Twickenham

Palin, Ralph, M. D. Newport, Shropshire
Palm, Dr. Newport
Parkin, Henry, M. D. Cawsand, Plymouth Dock
Parson, Mr. C. A. Godalmin
Paynter, J. W. Esq. Surgeon, Pembroke
Peacock, F. (M. D.) Darlington
Pedder, George, Esq. Member of the Royal College of Surgeons, Ryde, Isle of Wight
Percy, Dr. H. G.
Peterborough Dispensary, Northamptonshire
Pitt, Mr. Surgeon, Brighton
Poysers, Mr. Surg. Werkworth

Q.

Quead, Mr. Surgeon, Royal Navy
Quinton, Dr. William

R.

Ramadge, Francis, H. Member of the Royal College of Physicians, and Physician to the Infirmary for the cure of Asthma, Consumption, &c. Union St. Bishopsgate
Rann, Dr. Benborough, Oxfordshire
Ridge, Mr. Surgeon, Bridge-road, Lambeth
Roberts, Mr. John, Surgeon, Lothbury
Robinson, Dr. Princes-street, Stamford-street
Rocliffe, Dr. L. Easingwood, Yorkshire
Rogers, P. Esq. Member of the Royal College of Surgeons, Cornwall
Rogerson, Mr. John, Blackburn
Round, Mr. Cheque Office, Bank
Rowe, Matthew, Esq. Surgeon, Burton Crescent
Ruxton, John, Esq. Ensham Hall, Oxon

S.

Salmon, R. Esq. Wrickham Munket
Sandwich Medical Society
Schumadher, C. R. H. M. S. Dasher—To West Indies
Scott, James, M. D. Armagh, Ireland
Scott, P. N. Esq. Surgeon, Norwich
Scripp, Mr. Surgeon, Welsh Pool
Scudamore, Dr. E. Wye, Kent, 3 copies
Semple, Mr. Surgeon
Sexty, Mr. Surgeon, West Kent Militia, Maidstone
Seymour, Mr. J. G. Bishops Waltham
Sharpe, H. Esq. Surgeon, Milton, Suffolk
Shaw, Mr. George, Surgeon, Halifax, Yorkshire
Shand, Dr. Royal Navy, Cape of Good Hope
Shillitoe, Richard, Member of the Royal College of Surgeons in London, Hartford
Shillito, Mr. Charles, Surgeon, Putney
Simpson, Dr. Malton, Yorkshire
Simpson, Thomas, Esq. Surgeon, Knaresbro'
Simpson, Mr. H. Liddon, Norfolk
Smith, Dr. Ashby, Bloomsbury-square
Smith, Southwood, M.D. Licentiate of the Royal College of Physicians, London, Physician to the London Dispensary, Trinity-square
Smith, Mr. William Bartoe, Surgeon, Ludbury
Sprague, Mr. Surgeon, Kingston-upon-Thames
Spurgin, Mr. Thomas
Stephenson, Surgeon, 48th Regt.
Stevenson, John, Esq. Great Russell-street

Stratten, Samuel, M. D. Neath Hospital, Dublin
Stratton, Mr. Surgeon, Charlotte-street, Fitzroy-square
Surrage, T. L. Esq. Surgeon, Wincanton, Somersetshire
Sutherland, J. A.M. Surgeon, Southwold, Suffolk
Swain, Jas. Esq. Upper Charles-street, Northampton-square
Syder, John, M. D. Melton
Syder, Mr. C. M. Surgeon, Lancaster-street, Burton Crescent

T.

Taylor, Mr. Surgeon, Steyne, Brighton
Taylor, W. Esq. Surg. North Yarmouth
Terry, Henry, Esq. Surgeon, Northampton
Thomas, Dr. Cheltenham
Thomas, Mr. Isle of France
Thomas, Mr. Henry, Hebden Bridge, near Halifax, Yorkshire
Thomson, Henry Benwell, Esq. of the Royal College of Surgeons, and one of the Surgeons to the Kent Dispensary
Thomson, Jno. M.D. Edinburgh
Tolmie, James, Esq. Surg. Cambletown, Fort George, N. B.
Tommasini Giacomo, Bologna
Turner, Mr. Surgeon, Stanley Bridge, Ashton-under-line

U.

Urkwart, Mr. John D.

W.

Wake, Dr. York
Walker, Dr. Physician to the British Embassy, St. Petersburgh
Watling, Thomas, Esq. Member of the Royal College of Surgeons, Leominster
Watson, Mr. (Student) Edinburgh
Welsh, Mr. W. Surgeon, Plymouth Dock
West, Mr. Edward, Surgeon, Camelford, Cornwall
Williams, Mr. Robert, Surgeon, Corwen, Merionethshire
Wilson, Mr. Surg. Arbroath
Woodall, Mr. Robert, Member of the Worshipful Society of Apothecaries, Manchester

Y.

Young, Mr. Ploughman
York County Hospital I i rary
Young, Jas. Esq. Surg. Wells
Young, Mr. Samuel, Su geon, Reading
York, Charles Busto, Esq.

---

N. B. It is to be particularly remarked, that Subscribers are to procure the Journal in any way most convenient to themselves—that is, through the medium of their own booksellers, who are to order it from their London correspondents along with other periodicals, on the 1st days of JUNE —SEPTEMBER—DECEMBER—and MARCH ; at which periods it is most punctually published. Subscribers are requested to send their names early in each quarter, as the Journal closes fifteen days before publication day.

*Additional Subscribers since last Quarters*

Attree, William, Esq. Surgeon, Brighton
Beck, Mr. Edward, Jesus College Cambridge
Bloomfield, Dr. Woodbridge
Bradley, Mr. Joseph, Mytholm Royd, near Halifax, Yorkshire
Cochran, Dr. Madras Establishment
Davis, Mr. Surgeon, Andover, Hants
Dickson, Mr. Surgeon, H. M. S. Carnation, West Indies
Duke, Dr. Valentine, late Surgeon to H.M. Naval Hospital, Cape of Good Hope
Dumfries Medical Society
Editors of Edinburgh Medical and Surgical Journal, in exchange for Med. Chir. Rev.
Field, Henry, M.D. Black-rock, near Dublin
Freeman, Joseph, Esq. Spring Gardens
Freeman, R. Esq. Saxmundham
Goodwin, Mr. Surgeon, Hon. E. I. Comp. Service
Graham, Mr. Surgeon, Broad Street, Golden Square
Gwyn, R. H. Esq. Surgeon, Broseley, Salop
Hardy, Dr. C. H. Fellow of the Royal College of Physicians, Bath
Harris, Mr. Surg. Mark Lane
Harsant, Mr. C. Wrickham Munket
Hart, William, Esq. Surgeon, Dorking, Surrey
Ireland, Mr. Surgeon, Hart Street, Bloomsbury
Jackson, Mr. T. D.—To East Indies
Keegan, Dr. Halifax, Nova Scotia
King, R. C. Esq. Saxmundham
Lynn, G. D. Esq. Woodbridge
Mac Cabe, Dr. London
Manley, Dr. James R. New York (in exchange)
Norman, Charles James, Esq. To West Indies
Osborn, Alick, Esq. H. M. S. Leven—To West Africa
Padmore, Mr. Charles Henry, Surgeon, Twickenham
Peacock, F. (M.D.) Darlington
Quinton, Dr. William
Ridge, Mr. Surgeon, Bridge Road, Lambeth
Salmon, R. Esq. Wrickham Munket
Schumadher, C. R. H. M. S. Dasher—To West Indies
Scott, James, M. D. Armagh, Ireland
Scudamore, Dr. E. Wye, Kent, 3 copies
Sharpe, H. Esq. Surg. Milton, Suffolk.
Shaw, Mr. George, Surgeon, Halifax, Yorkshire
Simpson, Mr. H. Liddon, Norfolk
Smith, Dr. Ashby, Bloomsbury Square
Smith, Mr. William Bartoe, Surg. Ludbury
Stratton, Mr. Surgeon, Charlotte Street, Fitzroy Square
Stratton, Samuel, M. D. Neath Hospital, Dublin
Syder, John, M. D. Milton
Thomas, Mr. Henry, Hebden Bridge, near Halifax, Yorkshire
Urkwart, Mr. John D.
Williams, Mr. Robert, Surgeon, Corwen, Merionethshire
West, Mr. Edward, Surgeon, Camelford, Cornwall
Young, Mr. Samuel, Surgeon, Reading
York, Charles Busto, Esq.

### INDEX.

A.

Abdomen, gun-shot wounds of, by Baron Larrey .......... 260
Abortion, a species of ........ 416
Abscess behind the œsophagus. 518
Acids, their chemical qualities. 315
Acupuncture, Mr. Churchill on 431
Adams, Sir William, his claims 449
Adipocere, Dr. Ure on ........ 321
Alentego, med. topography of.. 589
Alibert, M. on Tinea Capitis .. 534
Alkalies, their properties ..... 316
Amaurosis .................. 378
——— Dr. Weller on...... 650
——— Mr. Stevenson on .. 788
Amputation at the hip-joint... 194
——— during the progress of gangrene .... 186
——— proper period for operating ....... 176
——— without ligature of arteries ........ 248
Andalusian fevers, Dr. Jackson 485
Aneurism of the groin......... 269
——— Sir Astley Cooper's pathology of ........ 743
——— Mr. Coates's case of.. 870
——— Mr. Salmon's case of. 876
Angina pectoris, causes of .... 565
Antigua, med. topography of.. 592
Apothecaries-Surgeon, report of 454
Arachnitis, Duchatelet and Martinet on .................. 695
Argus, No. 1. on medical conduct ...... ........ 222
——— No. 2. obstinate pervigilium .............. 224
——— No. 4. Sir Charlatan Glanderman ........ 224
Arsenic, Dr. Gregory on ...... 871
Arteries, inflammation of...... 50
——— wounds of .......... 266
——— on the vacuity of.... 443
Arteritis, Mr Golding's case of 439
——— case of ............ 683
Attraction, Dr. Ure on........ 316

B.

Bacot, Mr. on syphilis........ 598
Bell, Mr. Charles, on the nerves 809
Berezina, passage of, by the French ................ 245
Berlinghieri, Prof. on lithotomy 337
Bibliographical Record—p. 230
Biography of Dr. J. Gregory.... 216
Bladder, wounds of .......... 262
Blepharophthalmitis ........ 630
Blood-letting, in hæmorrhagic diseases .................. 396
Boyle, Mr. on Indian Cholera.. 85
Brain, erethism of, by Dr. Nicholl ............527 361
——— masked disease of...... 440
——— morbid anatomy of.... 559
——— on concussion and compression .................. 740
Bridlington epidemic, account of 203
Bronchocele, cured by iodine.. 322
——— Mr. Hutchison on 866
Broussais, M. on gastritis and enteritis .................. 1
Brunonianism in Italy........ 747

C.

Cadiz fevers, Dr Jackson on.. 485
Calculi renal, Mr. Earle on.... 436
——— Sir Astley Cooper on extraction of ................ 874
Calculous disorders, Dr. Prout on ...................... 98
Calhoun, Dr. on paralysis .... 279
Caloric, Dr. Ure on .......... 317
Campaign. Russian, in 1812.... 237
Cancer, the present modes of treating .................. 733
Cardiac diseases, M. Fodéré on 417
Carmichael, Mr. case of amputation at the hip-joint ...... 511
Cartilages, ulceration of...... 425
Cataract.................... 380
——— Dr. Weller on ...... 642
Cephalonia, plague at ........ 580
Chancre, Mr. Bacot's observations on .................. 608
Chemistry, Dr. Ure's dictionary of.................... 315
Chest, wounds of, by Baron Larrey ................ 257
——— pathological examination of .............. 562
——— Drs. Laennec and Forbes on .................... 795
Chevalier, Mr. Hunterian oration...................... 220

Children, bones of, bent...... 141
Chlorine, Sir H. Davy on...... 317
Cholera of India, Mr. Boyle on 85
Churchill, Mr. on Acupuncture 431
Cinchonine, chemical and medical properties of.......... 670
Clarke, Mr. Mansfield, on female diseases.............. 757
Clysma, article on, by M. Barbier.................... 348
Coates, Mr. case of aneurism.. 870
Colchicum seeds, where to be procured................. 437
Colleges of Physicians and Surgeons, their laws .......... 734
Conception, extra-uterine .... 425
Conjunctiva, diseases of ...... 373
Conquest, Dr. outlines of midwifery.................... 47
Constitutional symptoms of syphilis .................... 611
Contagion of plague, how long dormant................ 583
Contagion of plague, laws of.. 584
Cooper, Sir A. on hernia cerebri 251
——— his lectures...... 736
——— *removal of a tumour* 880
Cooper, Sir A. on extraction of calculi.................. 874
Corfu, plague at.......... .... 576
Cornea, diseases of .......... 374
Croton, oil of, a new old remedy 428
Cubebs, Mr. Jeffreys on, in gonorrhœa................... 271
Cynanche laryngea, severe cases of....................... 516

D.

Death, sudden ............... 389
Dew, Dr. Ure on ............ 318
Dewees, Dr. on gastrotomy.... 278
Diagnosis between hydrocephalus and worm-fever ........ 470
Dictionary of chemistry ...... 315
Diet, in indigestion .......... 297
Digestive organs, mucous membrane of .. 1
——— Dr. Thomas on 348
Doctrine, new Italian delineated 745
Drink, in indigestion ........ 298
Duchatelet, on arachnitis.... 695
Dunn, Mr. on amputation of the tarsus ..................... 873

E.

Ear, Mr. Swan on............. 873
Earle, Mr. H. on the urethra.. 858
——— on renal calculi.. 436
Economy, female, Dr. Power on 409
Editor's case of hæmato-scrofulous tumour .............. 514
Emetics in hysteria .......... 282
Emphysema, remarkable case of 238
——— idiopathic, cases of..................... 335
Epigastrium, contusions on.... 662
Erethism of the brain, Dr. Nicholl on .................. 361
Eruptive diseases, Dr. Robinson on ...................... 335
Erysipelas, Mr. James on .... 857
Etiology of indigestion........ 292
Eudiometer, a new one...... 319
Excrescence, cauliflower...... 778
Experiments on the digestion of rabbits .................. 311
Eye, Dr. Wellers's treatise on.. 626
Eyes, diseases of, Mr. Travers on......................369 105

F.

Falloon, Dr. on diseases of the heart ..................... 532
Farr, Mr. on cancer.......... 732
Femur, fracture, with separation of condyles................ 242
Ferguson, Dr. on marsh poison 585
Fever, Mr. H. Sandwith on.... 203
——— Dr. Reid on.......... 327
——— congestive, Prosper Alpinus on.................. 685
Fevers of Spain, Dr. Jackson on 485
Forbes, Dr. translation of Laennec ..................... 795
Forensic case, Mr. Chas. Bell's 561
——— medicine, Dr. Smith on ....................... 387
Frost-bites, treatment of...... 246
Fucus helminthocorton, Mr. Farr on .................. 732
Fumigation, sulphureous, Mr. Wallace on................ 570
Fungus hæmatodes retinæ .... 653

G.

Galvanic experiments on digestion.......... .......... 311
Gangrene, traumatic, amputation, during .... ........ 187
Gangrene, Dr. Grattan's case of 521
Gastritis, acute and chronic.... 6
Gastrotomy, Dr. Dewees on.... 278
Glaucoma, Dr. Weller on.... 644
Glysters, observations on...... 353
Golding, Dr. case of arteritis.. 439
Golis, Dr. on hydrocephalus acutus..................... 465
Gonorrhœa, Mr. Jeffrey's on.. 271
Gozo, plague at.............. 575
Grattan, Dr. case of gangrene.. 521
Gravel, Dr. Prout on......... 98
Gregory, Dr. on marasmus.... 866

Gregory, Dr. on malformation of the heart .................. 871
Gregory, Professor, his death.. 216
Guadiana, medical topography of........................ 588
Gun-shot wounds, Messrs. Guthrie, Hennen, Laurent, and Percy, on ................. 165

H.

Hæmorrhage internal, case of.. 668
Hancock, Dr. on pestilence.... 569
Harrison, Dr. his case of hæmorrhage ............ 334
——— Mr. on syphilitic eruptions ..................... 598
Head, on wounds of, by Baron Larrey.................... 249
Heart, diseases of, Dr. Reeder. 401
——— Dr. Fodéré. 417
——— Dr. Falloon. 532
Helminthocorton, or Corsican moss ...................... 732
Hepatic abscess, from wounds of head............. 253
——— functions, Drs. Pearson and M'Donnel on.......... 281
Hernia cerebri, treatment of.. 251
——— Mr. Shaw on.......... 553
Hip-joint, amputation at...... 194
——— operation, Mr. Carmichael ................... 511
Hordeolum, treatment of...... 383
Hordeolum.................. 632
Hunterian oration, Mr. Chevalier's ................. 220
Hutchison, Mr. on bronchocele 866
Hydatids of the uterus........ 774
Hydrocephalus acutus, Dr. Golis on ...... 465
——— Sir G. Blane on 669
Hydrophthalmia, Dr. Weller on 647
Hydrothorox, pathology of.... 803
Hypopion ................... 375
Hysteritis, Mr. Clarke on...... 763

I.

Iliac, external, propriety of tying ................. 270
——— artery, on the ligature of 556
Indigestion, Dr. Philip on.. 862 284
Inflammation of arteries...... 50
——— of the spinal arachnoid...... 724
——— Mr. James on.. 834
Intermittents, masked........ 450
Iodine, Dr. Coindet on, in bronchocele .................. 322
Ireland, Dr. case of emphysema 335
Iritis, Dr. Weller on.......... 648
Irritation, Sir Astley Cooper on 737
Ischuria, Sir Astley Cooper on.. 744
Italy, present state of medicine in......................... 745

J.

Jackson, Dr. on the Andalusian fevers ................... 485
James, Mr. on inflammation.. 834
Jeffreys, Mr. Henry, on cubebs 271

K.

Kennedy, Dr. J. case of arteritis 60
Kentucky, university of...... 219
Kerrison, Dr. on tic douloureux 63
Kinine, the medical properties of........................ 670

L.

Labour, difficult case of...... 438
Lacon, author of, remonstrated with ..................... 522
Lacrymal passage, obstruction of........................ 385
Laennec, Dr. on thoracic diseases ...................... 795
Larrey, Baron, his Russian campaign ................... 237
Lavemens, on the use of...... 353
Lectures, Sir Astley Cooper's.. 736
Leeching, observations on.... 357
Lendrick, Dr. case of poisoning 534
Lippitudo ................... 384
Lisbon, medical topography of 589
Lithotomy, new method of operating.......... 337
——— Mr. Martineau on 877
Liver-cough, Dr. Brooke's case of........................ 524
Lungs, inflammation of, from wounds of other parts...... 192

M.

M'Donnel, Dr. on the hepatic functions ................ 281
M'Keever, Dr. case of ruptured vagina...................... 530
Malta, plague at............ 574
Manual of anatomy, Mr. Shaw's 551
Marasmus, Dr. Gregory on.... 862
Marathia, plague at.......... 577
Marsh poison, Dr. Ferguson on 585
Martineau, Mr. on lithotomy.. 877
Medicine forensic, Dr. Smith on 387
Medicines, large doses of...... 752
Medico-chirurgical transactions 865
Medico-politico-theatrico-metaphysical Record .......... 274
Medulla spinalis, inflammation of........................ 429
Melena, Dr. Nicholl on...... 529
Membranes, false, in pleurisy.. 798

Menstruation, Dr. Power on.. 409
——— deficient ...... 759
Mercury, case of gangrene from 522
——— various authors on.. 598
Midwifery, Dr. Conquest's outlines of ........ 47
——— Dr. Ramsbotham on 141
Military surgery, Messrs. Hennen and Guthrie on ......... 165
Military surgery, Baron Larrey on ..................... 240
Monteath, Dr. translation of Weller on the eye........... 626
Mucous membrane of the digestive organs................. 1
Mucous discharges (white) of females .................. 758

N.

Nerves, on affections of...... 63
——— Mr. Chas. Bell on their functions.................... 809
Nervous sympathy, how produced .................... 295
Nervous system, Mr. Bell's remarks on ................... 566
Neuralgia, singular case of.... 426
Nicholl, Dr. elements of pathology .......... 133
——— on erethism of the brain........... 361
——— on erethism of the brain.......... 527
——— on melena ...... 529
Noia, plague at, in 1815...... 582

O.

Observations, practical, in midwifery........ 141
——— in medicine, &c. by Mr. T. Sandwith......... 395
O'Halloran on the Spanish fever 817
Omentum, protrusion of...... 261
Ophthalmic inflammation, cure of. 370
——— suppurative........ 372
——— hospital extinct.. 448
Opiologia; or confessions of opium-eaters.............. 881
Opium, particular preparation of.................. 219
——— its effects on the human system................... 883
Osborn, Dr. on the urine...... 41
Osteo-sarcomatous tumour.... 511
Os uteri, case of separation, by Mr. Scott ................ 876
Oxymuriate of mercury, swallowed .................... 534

P.

Palpitations of the heart, Dr. Fodéré on ................. 417
Panckouckerie ............... 447
Paralysis, tourniquets in...... 279
Par vagum, remarks on ...... 566
Patella, dislocation of ........ 682
Pathology of the urine........ 41
——— Dr. Nicholl's elements of ............... 133
——— of indigestion ...... 296
Percussion in chronic pleurisy.. 800
Pericardiac inflammation...... 395
Perineum, ruptured........... 684
Peritoneal inflammation, Mr. Guthrie on................ 171
Perivoli, plague at .......... 578
Pestilence, Dr. Hancock on.... 665
Phagedena, Mr. Welbank on .. 875
Philadelphia Journal of Medical and Physical Sciences ...... 275
Philip, Dr. on indigestion.. 862 284
Phlebitis, curious case of...... 453
Phlegmasies chroniques, M. Broussais on............... 1
Physiological experiments on digestion.................. 311
Pierson, Dr. on the hepatic functions ................. 281
Pinel, M. on the medulla spinalis ...................... 429
Plague, Mr. Tully and Dr Hancock on ... .............. 569
Pleurisy, pathology of........ 796
——— chronic ............ 800
Plumbe, Mr. on porrigo ...... 534
Pneumo-thorax ............... 804
Polypi of the heart... ........ 564
Pomegranate bark, Mr. Breton on ...................... 872
Poultices, evaporating, in gunshot wounds ............... 176
Power, Dr. on menstruation, abortion, &c... ........... 409
Price, Mr. case of sudden death 870
Prince Ruperts, port of...... 591
Protracted labours, remarks on 48
Prout, Dr. on gravel, calculus, &c....................... 98
Pterygium .................... 374
——— .................. 641
Pupils, artificial, Dr. Weller on 449

Q.

Quackery, on the suppression of 734
Quarantine, Mr. Tully's remarks on ................ 583

R.

Rabbits, Dr. Philip's experiments on ................ 310

Ramsbotham, Dr. on midwifery 141
Recto-vesical operation ...... 337
Reeder, Dr. on diseases of the heart........... 401
———— letter to the reviewers ................. 686
Reid, Dr. on fever............. 327
Repletion, fatal species of fever from ..................... 247
Respiration, remarks of Dr. Ure on ..................... 325
Respiratory nerves, Mr. C. Bell on ..................... 809
Ringworm of the scalp, works on ..................... 534
Robinson, Dr. on eruptive diseases ..................... 335

S.

St. Lucie, med. topography of.. 590
Sandwith, Mr. Humphrey, on fever........ 203
———— Thomas, observations in medicine and surgery..................... 395
Sangsue, article on, by M. Merat 348
Sarcocele, M. Maunoir on.... 682
Sclerotitis ................. 376
Scott, Mr. on separation of os uteri ..................... 876
Scourge of Venus and Mercury 597
Scriblomania.. .............. 228
Secale cornutum, or ergot of rye 442
Shoulder joint, terrible laceration of ................. 240
Sintelaer, Dr. on venereal diseases ..................... 597
Smith, Dr. on emetics........ 282
———— John Gordon, on forensic medicine............. 387
Spain, fever of, Mr. O'Halloran on ................. 817
———— degraded state of medicine in..................... 820
Spinal system, Dr. Reid on.... 331
———— arachnitis ............. 724
Spleen, case of hæmorrhage from ..................... 334
Staphyloma ................. 376
———— Dr. Weller on.... 654
Stevenson, Mr. on amaurosis.. 788
Stimulants and contra-stimulants ..................... 749
Stomach, unsuspected ulcer of 446
Strumous ophthalmia ........ 371
Subclavian artery, wound of.. 267
Surgical observations, by Mr. T. Sandwith ................ 398
Swan, Mr. on affections of nerves 63
———— on the ear.......... 873
Swediaur, Dr. on venereal complaints..................... 598
Syder, Mr. Myngay, his notes of lectures................. 736
Sympathy, observations on.... 295
Symptomatology of indigestion 284
Syphilis, Dr. Thomson on the origin of................. 616

T.

Tarsus, Mr. Dunn on amputation of..................... 873
Tendo Achillis, rupture of, treatment of ................. 740
Tetanus, successful case of, Mr. Burmester................. 876
Thomas, Dr. on the digestive organs..................... 548
Thomson, Dr. J. on mercury .. 598
Thoracic diseases, Drs. Laennec and Forbes on ............ 795
Throat, wounds of............ 256
Tibia, amputation at the head of 243
Tic douloureux, Dr. Kerrison on 63
———— Mr. Swan on.. 68
Tincture of poppy, Dr. Wilson on ..................... 669
Tommassini on the new Italian doctrines................. 745
Tracheotomy, cases of, Mr. Carmichael...... 516
———— case of, by Mr. Porter..................... 878
Transactions of the New York medical society............ 275
Transactions of the Irish College, vol. iii. .............. 327
Transactions of the King's and Queen's College............ 511
Travers, Mr. on diseases of the eye ..................369 105
Treatment of indigestion...... 297
Trephine, seldom necessary.... 250
Tully, Mr. on plague.......... 569
Tumour, immense, removed by Sir A. Cooper.............. 880

U.

Ulcer in the stomach, unsuspected..................... 446
Urethra, muscular fibres in?.. 555
———— Mr. Earle on........ 858
Urine, on the physiology and pathology of ........ 41
———— involuntary discharge of 788
Uterine disease, obscure case of 455

V.

Vacca, Professor, on recto-vesical operation.. ...... ...... 337

Vagina, ruptured, case of...... 530
Ventriloquism .............. 219
Villers, Dr. on tic douloureux.. 63
Viscera, morbid anatomy of.... 553

W.

Wallace, Mr. on sulphureous fumigation... ............ 507
Water-stroke, (wasserschlag) Dr. Golis on .............. 466
Watery discharge from the uterus ...................... 777
Weller, Dr. on diseases of the eye ...................... 626
Wilbank, Mr. on sloughing phagedena..................... 875
Wounds, gun-shot, Messrs. Guthrie, Hennen, Laurent, and Percy on.............. 165
Wounds of head, Baron Larrey on ............... 249
——— in dissecting, treatment of ............... 552
——— from dissection, treatment of ................. 739

Y.

Yellow fever, Dr. Ferguson on 594
——— Dr. Jackson on.. 485
——— Mr. O'Halloran on ..................... 817

P.S. We have to regret that several Subscribers' names, arrived after the Journal was closed, and when we had no other place to mention the circumstance than this.

### ERRATA.

No. 5, page 1, line 9 from bot. *for* complimentaire *read* complementaire
——— page 55, line 19 from bot. *for* λογος, *read* νοσος.
No. 7, page 598, *for* Henry, *read* John Thomson.
——8, page 747, *for* Lio Scozzes, *read* Lo Scozzes.

*Directions to the Binder.*

The Tables of Contents are to be taken from the four Numbers published, and to be bound up with the Volume after the Title Page. They are paged for that purpose.

